# Supplementary figures and images for: Metagenomic insights into surface water microbial communities of a South Asian mangrove ecosystem
Source: PeerJ. 2022 May 9;10:e13169. doi: 10.7717/peerj.13169 (PMC9097664; doi:10.7717/peerj.13169)

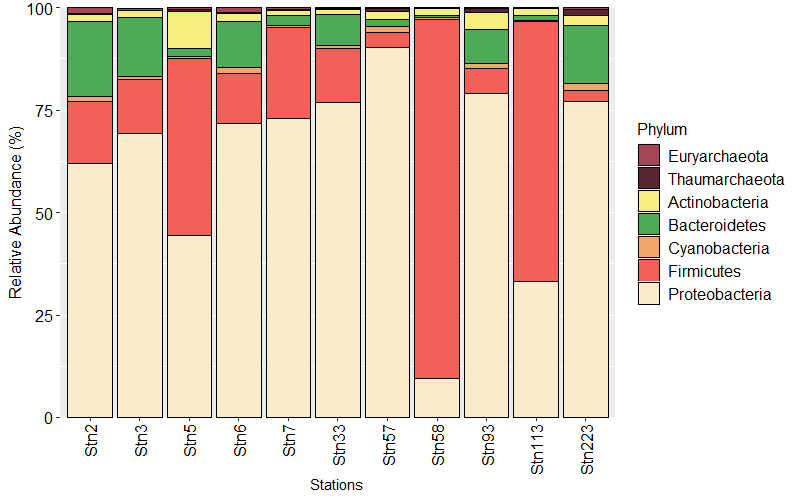

Supplement: Supplemental Information 1 [file peerj-10-13169-s001.tiff]

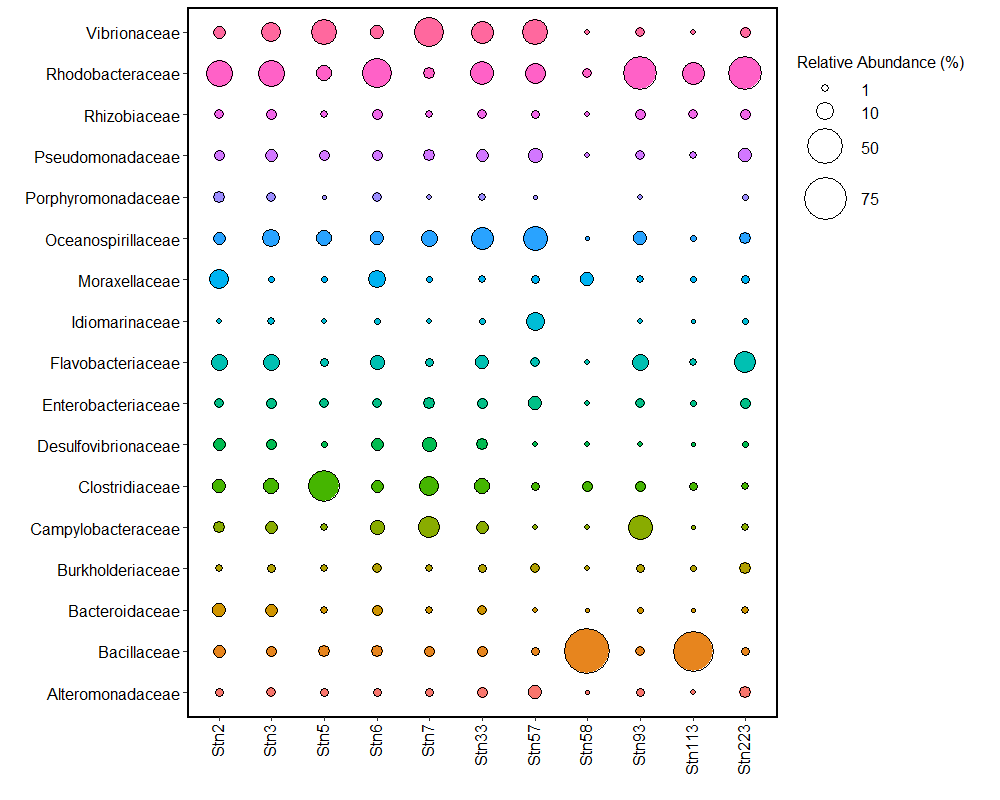

Supplement: Supplemental Information 2 — The size of the circle denotes the relative percentage of the bacterioplankton family within the entire bacterioplankton community. [file peerj-10-13169-s002.tiff]

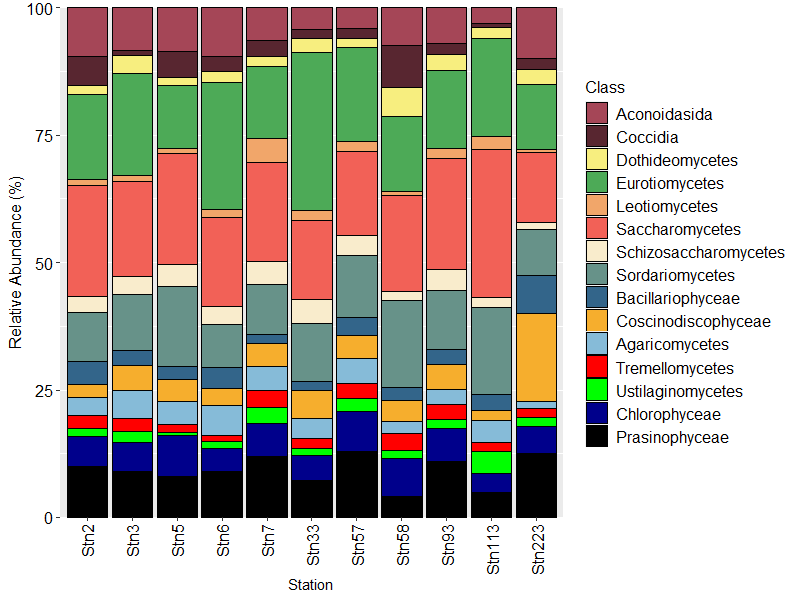

Supplement: Supplemental Information 3 [file peerj-10-13169-s003.tiff]

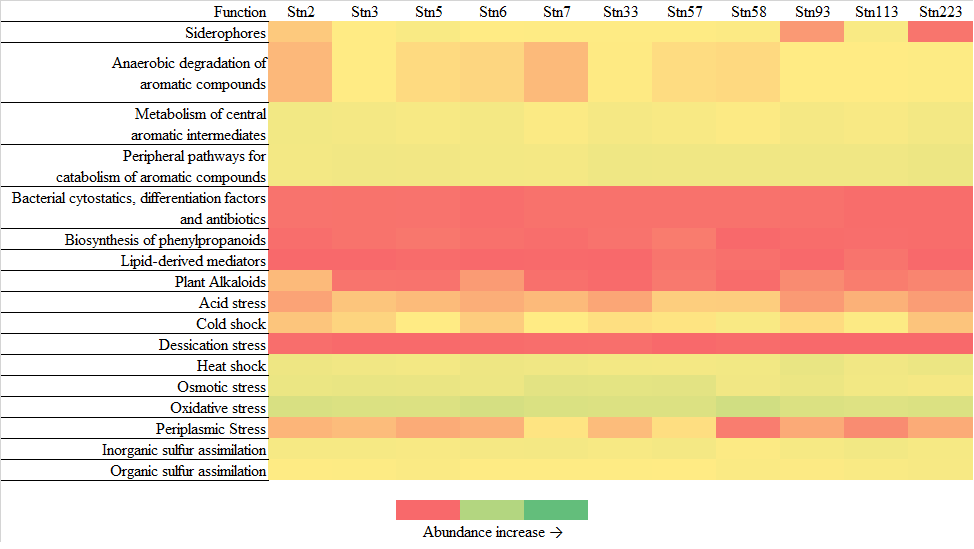

Supplement: Supplemental Information 4 — Only genes involved in specialized functions such as adaptation and secondary metabolite production are shown. [file peerj-10-13169-s004.tif]

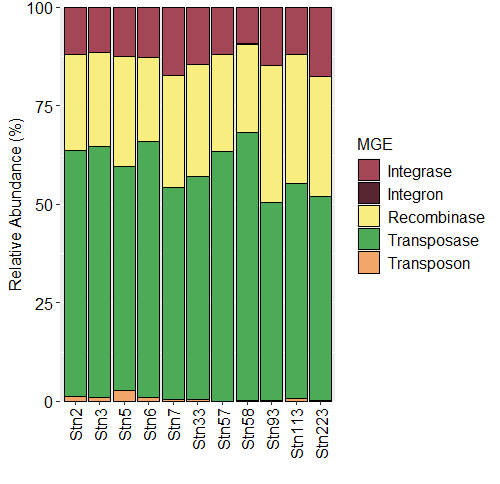

Supplement: Supplemental Information 5 [file peerj-10-13169-s005.tiff]

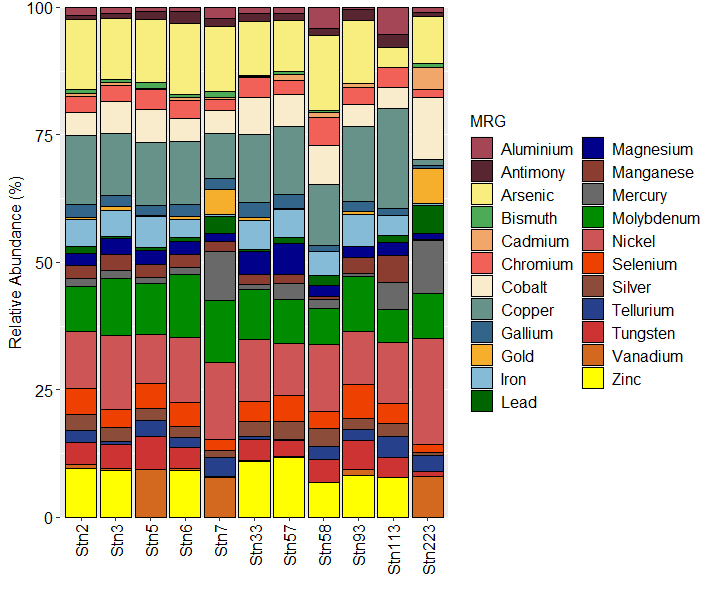

Supplement: Supplemental Information 6 [file peerj-10-13169-s006.tiff]
