# Supplementary material for: Metagenomic insights into surface water microbial communities of a South Asian mangrove ecosystem
Source: PeerJ. 2022 May 9;10:e13169. doi: 10.7717/peerj.13169 (PMC9097664; doi:10.7717/peerj.13169)
Supplement: Supplemental Information 7 [file peerj-10-13169-s007.docx]

Table S1: Environmental parameters and dissolved nutrient concentrations measured at the studied stations in June, 2019

| Station | Latitude | Longitude | Dissolved Ammonium (µM) | Dissolved Nitrate  (µM) | Dissolved *o*-phosphate (µM) | Dissolved Silicate (µM) | AT (˚C) | SWT (˚C) | Salinity | pH | TDS (ppm) | EC (µS/cm) | DO (mg/L) | Secchi depth (cm) |  |
| --- | --- | --- | --- | --- | --- | --- | --- | --- | --- | --- | --- | --- | --- | --- | --- |
|  |  |  |  |  |  |  |  |  |  |  |  |  |  |  |  |
|  |  |  |  |  |  |  |  |  |  |  |  |  |  |  |  |
| SBR_Stn2 | 21.9854 | 88.63263 | 0.05 | 33.23 | 0.51 | 38.43 | 30.4 | 31 | 30.5 | 7.57 | 17440 | 33600 | 12.8 | 10 |  |
| SBR_Stn3 | 21.9755 | 88.63118 | 1 | 33.75 | 0.98 | 22.86 | 30 | 31.1 | 30.5 | 7.5 | 16200 | 32600 | 14.3 | 10 |  |
| SBR_Stn5 | 22.0052 | 88.64747 | 1 | 38 | 0.49 | 27.14 | 33.2 | 31.5 | 30.3 | 7.54 | 15800 | 31200 | 7.4 | 6.5 |  |
| SBR_Stn6 | 22.0051 | 88.65042 | 3 | 37.5 | 0 | 0 | 32.5 | 31.4 | 30.2 | 7.5 | 16520 | 33200 | 10.3 | 12 |  |
| SBR_Stn7 | 22.004 | 88.65244 | 2 | 39.75 | 0.49 | 11.79 | 30.8 | 31.1 | 30.4 | 7.5 | 16320 | 32720 | 10.5 | 9.6 |  |
| SBR_Stn33 | 21.9876 | 88.59597 | 1 | 47.25 | 0.49 | 0 | 29.4 | 31.6 | 29.3 | 7.47 | 17800 | 35720 | 6.6 | 7.5 |  |
| SBR_Stn57 | 21.8948 | 88.57629 | 6 | 41.75 | 0.98 | 0 | 26.8 | 30 | 29.3 | 7.54 | 14000 | 28000 | 7.8 | 6 |  |
| SBR_Stn58 | 21.8898 | 88.57586 | 2 | 33 | 0.49 | 17.86 | 27.2 | 30.4 | 29.6 | 7.54 | 14520 | 28600 | 7.5 | 6.5 |  |
| SBR_Stn93 | 21.7822 | 88.56316 | 0 | 40.5 | 0.73 | 43.93 | 29.9 | 29.8 | 24.9 | 7.38 | 9320 | 18400 | 7.36 | 8 |  |
| SBR_Stn113 | 21.7746 | 88.58857 | 1 | 34.5 | 1.46 | 13.21 | 31.3 | 31.7 | 28.8 | 7.53 | 12600 | 24640 | 7.16 | 13.5 |  |
| SBR_Stn223 | 21.5706 | 88.50754 | 2 | 33.25 | 1.71 | 17.14 | 29.4 | 29.7 | 24.6 | 7.29 | 15040 | 29720 | 7.16 | 16.3 |  |
