# Supplementary material for: Metagenomic insights into surface water microbial communities of a South Asian mangrove ecosystem
Source: PeerJ. 2022 May 9;10:e13169. doi: 10.7717/peerj.13169 (PMC9097664; doi:10.7717/peerj.13169)
Supplement: Supplemental Information 8 [file peerj-10-13169-s008.docx]

| Station | Total number  of reads | Number of sequences of rRNA genes | Number of Protein sequences with known function | Number of Protein sequences with unknown function | Sequences  affiliated  to ARG | Sequences  affiliated  to MGE | Sequences  affiliated  to MRG |
| --- | --- | --- | --- | --- | --- | --- | --- |
| SBR_Stn2 | 517176 | 845 | 100506 | 190678 | 844 | 1453 | 957 |
| SBR_Stn3 | 668660 | 954 | 109748 | 258982 | 838 | 1236 | 943 |
| SBR_Stn5 | 570433 | 1185 | 126665 | 182079 | 1239 | 1892 | 1036 |
| SBR_Stn6 | 445835 | 822 | 111000 | 124256 | 989 | 1697 | 1090 |
| SBR_Stn7 | 650670 | 1370 | 151518 | 209572 | 725 | 890 | 729 |
| SBR_Stn33 | 542884 | 1254 | 137843 | 167466 | 1421 | 1592 | 1496 |
| SBR_Stn57 | 609654 | 1895 | 175143 | 169648 | 1721 | 2703 | 1880 |
| SBR_Stn58 | 346884 | 1197 | 108571 | 77638 | 1457 | 2233 | 1156 |
| SBR_Stn93 | 424974 | 2648 | 325488 | 96838 | 1658 | 2103 | 1917 |
| SBR_Stn113 | 374471 | 4393 | 344268 | 25810 | 112 | 134 | 85 |
| SBR_Stn223 | 442509 | 489 | 101042 | 138462 | 693 | 1296 | 814 |

Table S2: Details of sequences affiliated to types of genes identified from the

sampling stations
